# Molecular taxonomy of *Dunaliella *(Chlorophyceae), with a special focus on *D. salina*: ITS2 sequences revisited with an extensive geographical sampling

**DOI:** 10.1186/2046-9063-8-2

**Published:** 2012-01-30

**Authors:** Patrícia Assunção, Ruth Jaén-Molina, Juli Caujapé-Castells, Adelina de la Jara, Laura Carmona, Karen Freijanes, Héctor Mendoza

**Affiliations:** 1 Departamento de Biotecnología. División de Investigación y Desarrollo Tecnológico. Instituto Tecnológico de Canarias (ITC). Pozo Izquierdo, 35119 Sta. Lucía, Canary Islands, Spain; 2 Departamento de Biodiversidad Molecular y Banco de ADN, Jardín Botánico Canario "Viera y Clavijo"-Unidad Asociada CSIC, Apartado de correos 14 de Tafira Alta, 35017 Las Palmas de Gran Canaria, Canary Islands, Spain

**Keywords:** Canary Islands, Compensatory Base Changes, *Dunaliella salina*, Internal Transcribed Spacer, Saltworks, Taxonomy

## Abstract

We used an ITS2 primary and secondary structure and Compensatory Base Changes (CBCs) analyses on new French and Spanish *Dunallela salina *strains to investigate their phylogenetic position and taxonomic status within the genus *Dunaliella*. Our analyses show a great diversity within *D. salina *(with only some clades not statistically supported) and reveal considerable genetic diversity and structure within *Dunaliella*, although the CBC analysis did not bolster the existence of different biological groups within this taxon. The ITS2 sequences of the new Spanish and French *D. salina *strains were very similar except for two of them: ITC5105 "Janubio" from Spain and ITC5119 from France. Although the Spanish one had a unique ITS2 sequence profile and the phylogenetic tree indicates that this strain can represent a new species, this hypothesis was not confirmed by CBCs, and clarification of its taxonomic status requires further investigation with new data. Overall, the use of CBCs to define species boundaries within *Dunaliella *was not conclusive in some cases, and the ITS2 region does not contain a geographical signal overall.

## Background

The Internal Transcribed Spacer 2 (ITS2) of the nuclear rDNA cistron is one of the most frequently used regions for phylogenetic analysis in algae [1-3]. Although its application in deep taxonomic levels was initially limited to comparisons of genera within the same family owing to uncertainties in alignment at higher taxonomic levels, the analysis of its secondary structure has provided key solutions to this problem [4]. Thus, the use of an ITS2 secondary structure improves sequence alignments, resulting in a higher robustness and accuracy of phylogenetic reconstructions [5] and providing help to distinguish species [6]. Furthermore, an automatic approach to analysis is possible [7], as a pipeline consisting of the ITS2 Database (annotation/structure prediction), 4SALE (alignment), ProfDistS (inferring phylogenies) and the CBCAnalyzer (distinguishing species) have recently become available (http://its2.bioapps.biozentrum.uni-wuerzburg.de/?about).

In *Dunaliella *(Chlorophyceae), the use of ITS2 secondary structure for phylogenetic analysis has a long tradition [8-13]. The genus *Dunaliella *comprises twenty-eight recognized species separated in two subgenera, *Pascheria *(which contains the freshwater species), and *Dunaliella *(grouping the marine species); the latter is further subdivided into four sections: *Tertiolecta, Dunaliella, Viridis *and *Peirceinae *[11]. The species ascribed to these four sections occur in a wide range of marine habitats such as oceans, brine lakes, salt marshes, salt lagoons and salt water dishes near the sea [14], being *Dunaliella salina *Teodoresco (section *Dunaliella*) the most studied one. *Dunaliella salina*, is the most halotolerant eukaryotic photosynthetic organism known to date [14,15] since it shows a remarkable degree of adaptation to a variety of salt concentrations and it accumulates large amounts of carotenes under extremely stressful conditions such as high salinity, low nitrogen levels, and high solar radiation [14]. Nowadays, it is the best commercial source of natural β-carotene [14,16], and it also stands out as a source of glycerol [17].

One of the aspects of *D. salina *that have most intrigued researchers is the enormous variability within strains regarding its geographic, physiological, and morphological characteristics [18-24]. Recent phylogenetic analyses of ITS1+ITS2 combined with the analysis of the ITS2 secondary structure of *D. salina *strains have also revealed a high intraspecific variation [8-11,25].

The high genetic diversity detected in *D. salina*, its morphological plasticity, and the restricted geographical sampling used in all scientific publications to date have hindered taxonomic elucidation in this taxon. Our objectives are (1) to use the ITS2 sequences and secondary structure analysis in a thorough geographic and taxonomic representation of the genus *Dunaliella *and particularly *D. salina *(including new Spanish and French strains) to improve understanding of the complex phylogenetic structure in this taxon, (2) to study the relationship of the new strains with the *Dunaliella *sequences available at the ITS2 Database and/or at GenBank [26]; (3) to investigate if the Compensatory Base Changes (CBCs) analyses could elucidate the species concept in *Dunaliella*, and reveal potentially new species; and (4) to test if *D. salina *ITC5105 "Janubio" could be considered a new species.

## Results

**Figure 1:**
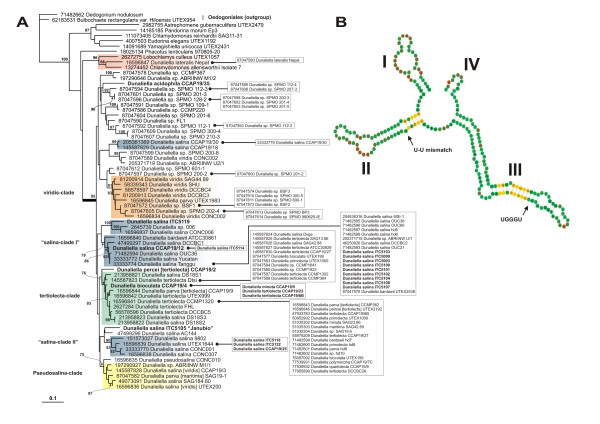
**Sequence-structure tree and consensus structure of ITS2**. (A) Sequence-structure Neighbor-Joining tree obtained by ProfDistS and supporting bootstrap values (100 replicates). Strains on the right written within squares have identical ITS2 sequences. Strains written in bold were sequenced in this study. All strains information can be found in Supplementary Table 1 (B) Consensus structure (51%) for all ITS2 sequences obtained from the complete multiple sequence-structure alignment without gaps. Helices are numbered I-IV. Sequence conservation is indicated from red (not conserved) to green (conserved). Nucleotides which are 100% conserved in all sequences are written as A, U, G or C. Nucleotide bonds which are 100% conserved throughout the alignment are marked in yellow. The figure was generated with 4SALE [41].

The ITS2 primary and secondary structure phylogenetic analysis of all the *Dunaliella *sequences available in the ITS2 Database plus the new sequences revealed great heterogeneity, although some of the clades were not statistically supported (Figure 1). No phylogenetic relationship is supported between the two *Dunaliella *freshwater species, since *D. lateralis *was positioned outside the *Dunaliella *subgenus, while *D. acidophila *was positioned within this subgenus.

The strains positioned in the tertiolecta-clade represent species that belong to different traditionally accepted sections: section *Tertiolecta *(*D. tertiolecta, D. primolecta, D. quartolecta, D. polymorpha)*, section *Viridis *(*D. bioculata, D. minuta*), section *Dunaliella *(*D. salina, D. parva*), and section *Peircei *(*D. percei*). The majority of these strains had an exact ITS2 sequence (Figure 1). Most *D. viridis *strains sampled were positioned in a single clade; however, some strains (UTEX200, CCAP19/3) were positioned in a different clade together with *D. pseudosalina *CONC010 (pseudosalina-clade), but without statistical support.

The majority of the *D. salina *strains were distributed in two different clades (salina-clade-I and salina-clade II), positioned between the tertiolecta sub-clades, whereas two *D. salina *strains (CCAP19/30, CCAP19/18) were positioned together in a different clade. Only two of the new ITS2 *D. salina *sequences, ITC5119 ("salina-clade-I") and *D. salina *ITC5105 "Janubio" (salina-clade II), had a unique ITS2 sequence profile (Figure 1). The CBC analysis of the ITS2 secondary structure showed that there is at least one CBC between "Janubio" and the other *D. salina *strains analyzed (see Additional file 1), except *D. salina *CCAP19/30 and CCAP19/18; however these two strains were phylogenetically distant to Janubio.

The taxonomic identification of the *Dunaliella *sp. strains (below the *D. acidophila *strain in Figure 1) was not possible because they did not match with any known *Dunaliella *species. Despite the phylogenetic tree indicates that this group may indeed represent a new species; the analysis of the CBC did not confirm these results (Additional file 1).

We observed a lack of CBCs in some strains when we compared species in different clades: viridis *vs *salina, viridis *vs *tertiolecta, salinas *vs *tertiolecta, salina *vs *salina, etc (Additional File 1). Also, the CBC analysis of the different species within the subgenus *Dunaliella *for the confirmation of the species boundaries was not conclusive in some cases (Additional file 1).

The phylogenetic results of the ITS2 sequences in this study, and previous confirmed identification of some *Dunaliella *strains (see Table 1), allow us to suggest the re-identification of some strains (Table 1).

**Table 1:** Taxonomic classification, Culture Collection, Geographic Origin and GenBank acession numbers of the strains included in this study.

Former classification	New suggested classification [Reference] and comments	Culture Collection	Geographic Origin	Isolator (Date)	GenBank acession number	GenBank identifier
Subgenus *Pascheria *(Freshwater species)						
*Dunaliella lateralis *Pascher &**Jahoda**			Nepal		AF313445	16596847
			Nepal		DQ377089	87047580
*Dunaliella acidophila *(Kalina) Massyuk		CCAP19/35	Freshwater; acidic sulphurous pool, Pisciarelli, Naples, Italy	Albertano (1981)	HM060646	
Subgenus *Dunaliella*						
Section *Tertiolecta *(Marine species. Optimum salinity < 6% NaCl)						
*Dunaliella tertiolecta *Butcher		CCAP19/6B	Brackish; Oslo Fjord, Norway	Foyn (1928 or earlier)	HM243579*	
		CCAP19/6B	Brackish; Oslo Fjord, Norway	Foyn (1928 or earlier)	AY572957	47933783
		CCAP19/27			EF473748	145587830
		CCAP19/27			AY654300	55979209
		Dtsi	Italy: Venezia		EF473730	145587823
		UTEX999	Norway: Oslofjord		AF313435	16596842
		CCMP1320	Salt flat. USA?		AF313433	16596841
		CCMP1302	Salt flat. USA?		DQ377096	87047587
		CCMP364	Salt flat. USA?		DQ377097	87047588
		FHL			DSU66956	2627284
		DCCBC5			AY686684	56578596
		SAG13.86	Norway: Oslofjord		EF473738	145587825
		ATCC30929	United Kingdom: Plymouth		EF473742	145587827
		DCCBC26			DQ224338	77955899
*Dunaliella quartolecta *Butcher	*Dunaliella tertiolecta [this study]*	CCAP19/8	Marine; Southampton, Hampshire, England	Butcher (1953)	DQ157054	77539932
*Dunaliella primolecta *Butcher	*Dunaliella tertiolecta [this study]*	UTEX1000	English Channel, Plymouth, Devon, England	Gross (1936)	AY582942	50952902
		UTEX1000	English Channel, Plymouth, Devon, England	Gross (1936)	DQ377092	87047583
	*Dunaliella tertiolecta [this study]*	hd8	China?		DQ116745	71482600
*Dunaliella polymorpha *Butcher	*Dunaliella tertiolecta [this study]*	CCAP19/7C	Brackish; River Crouch, Essex, England	Butcher (1954)	DQ157053	77539931
*Dunaliella marítima *Butcher	*Dunaliella tertiolecta [this study]*	SAG42.89			AY582086	51035303
Section *Dunaliella *(Halophilic species. Optimum salinity > 6% NaCl. Accumulates carotenes)						
*Dunaliella salina *Teodoresco		CCAP19/18	Hypersaline; Hypersaline brines, Hutt Lagoon, Western Australia	Kaethner (1982)	AF546098	33333776
		CCAP19/18	Hypersaline; Hypersaline brines, Hutt Lagoon, Western Australia	Kaethner (1982)	EF473746	145587829
		CCAP19/25			HM140783	
		UTEX1644	Point Colorado Salinas; La Paz, Baja California, Mexico	Loeblich (1967)	AF313429	16596839
		CONC006	Salar de Atacama, Chile	(1990)	AF313425	16596837
		CONC001	Laguna La Rinconada, Chile		AF546092	33333770
		CONC007	Salar de Atacama, Chile	(1990)	AF313427	16596838
		DCCBC1	Lake Tyrell, Victoria, Australia	Polle	AY549442	47499297
		DCCBC2	South Korea		AY512973	46250926
		hd6	Israel		DQ116743	71482598
			Yucatan, Mexico		AF546094	33333772
			Tanggu, China		AF546096	33333774
		AC144	Tunisia, North Africa		AY549441	47499296
	*Dunaliella viridis [this study]*	184.80			AY577766	49073091
		OUC66 "hd4"	China	(2005) ?	DQ116741	71482596
		OUC38 "hd3"	China	(2005) ?	DQ116740	71482595
		OUC36 "hd2"	China	(2005) ?	DQ116739	71482594
		OUC21 "hd1"	China	(2005) ?	DQ116738	71482593
		9802	China ?	(2007) ?	EF695405	151573027
		"hd5"	Inner-Mongolia	(2005)	DQ116742	71482597
	*Dunaliella tertiolecta [this study]*	DS18S1	Mexico?		FJ360756	213958821
	*Dunaliella tertiolecta [this study]*	DS18S2	Mexico?		FJ360757	213958822
	*Dunaliella tertiolecta [this study]*	DS18S3	Mexico?		FJ360758	213958823
	*Dunaliella tertiolecta [this study]*	Dsge	Belgium: Gent		EF473732	145587824
	*Dunaliella viridis *[ 9, 10, 27]	CCAP 19/3	Brackish; dirty salt lake, Soviet Union	Mainx	EF473744	145587828
	*Dunaliella viridis *[ 9, 10, 27]	UTEX200	Brackish; dirty salt lake, Soviet Union	Mainx	AF313423	16596836
		MSI-1			GQ337903	254838316
		ITC5100	Vargas, Gran Canaria, Spain	de la Jara & Mendoza (2005)	HM035353*	
		ITC5101	Punta, Gran Canaria, Spain	de la Jara & Mendoza (2005)	HM035354*	
		ITC5102	Tenefé, Gran Canaria, Spain	de la Jara & Mendoza (2005)	HM035355*	
		ITC5103	Rio, Lanzarote, Spain	de la Jara & Mendoza (2005)	HM035356*	
		ITC5104	Guatiza, Lanzarote, Spain	de la Jara & Mendoza (2005)	HM035357*	
		ITC5105	Janubio, Lanzarote, Spain	de la Jara & Mendoza (2005)	HM035346*	
		ITC5106	Carmen (Majo), Fuerteventura, Spain	Mendoza & Trujillano (2003)	HM035358*	
		ITC5107	Añana, Álava, Spain	de la Jara & Mendoza (2005)	HM035359*	
		ITC5118	île de Ré (01), France	Carmona & Mendoza (2006)	HM035348*	
		ITC5122	île de Ré (05), France	Carmona & Mendoza (2006)	HM035347*	
		ITC5114	La Tapa, Cádiz, Spain	de la Jara & Mendoza (2007)	HM035350*	
		ITC5119	île de Ré (02), France	Carmona & Mendoza (2006)	HM035349*	
	Aliquot of *Dunaliella salina *BCA421	ITC5003	Tenefe, Gran Canaria, Spain	Mendoza (1992)	HM035352*	
*Dunaliella bardawil *nomen nudum Ben-Amotz & Avron	*Dunaliella salina *CCAP 19/30 [27], obtained from Dr. Joao Varela (Faro, Portugal)	ITC5000	Marine; salt pond, near Bardawil lagoon, North Sinai, Israel	Ben-Amotz & Avron (1976).	HM035351*	
	*Dunaliella salina *[27]. Reinstated from SAG on April 1996	CCAP19/30	Marine; salt pond near Bardawil lagoon, North Sinai, Israel	Ben-Amotz & Avron (1976).	EU932917	205361369
	*Dunaliella salina *[27]	ATCC30861	Marine; salt pond near Bardawil lagoon, North Sinai, Israel	Ben-Amotz & Avron (1976)	AF313431	16596840
	*Dunaliella salina *[27]	UTEX2538	Marine; salt pond near Bardawil lagoon, North Sinai, Israel	Ben-Amotz & Avron (1978)	DQ377085	87047576
	*Dunaliella salina *[27], *Dunaliella tertiolecta [this study]*	SAG42.88	Marine; salt pond, near. Bardawil lagoon, North Sinai, Israel	Ben-Amotz & Avron (1976)	EF473741	
	*Dunaliella tertiolecta [this study]*	hd7	China?		DQ116744	71482599
*Dunaliella parva *Lerche	*Dunaliella viridis *[9-11]	UTEX1983	Dead Sea	(1973)	AF313441	16596845
	*Dunaliella tertiolecta *[10,11]*Dunaliella quartolecta *[27]	CCAP19/9	Brackish; salt marsh, Northey Island, Essex, England	Butcher (1956)	AF313439	16596844
	*Dunaliella tertiolecta *[10,11]	CCMP362		Gold	AF313437	16596843
	*Dunaliella marítima *[27], *Dunaliella viridis [this study]*	SAG19-1	Marine: Lacul Sarat, Romania	Lerche (Before 1938)	DQ377091	87047582
	*Dunaliella tertiolecta [this study]*	hd9	China?		DQ116746	71482601
*Dunaliella pseudosalina *Massyuk & Radchenko		CONC010	Salar de Atacama, Chile		AF313421	16596835
Section *Viridis *(Halophilic species. Optimum salinity > 6% NaCl. Cells always green. Do not accumulate carotenes. Cells radially symmetrical)						
*Dunaliella minuta *Lerche	*Dunaliella tertiolecta [this study]*	CCAP19/5	Marine; sand and sea water, Roscoff, France	Jowett (1967)	HM035345*	
	*Dunaliella tertiolecta [this study]*	SAG23.86			AY582085	51035302
*Dunaliella bioculata *Butcher	*Dunaliella tertiolecta [this study]*	CCAP19/4	Brackish; salt lake, Soviet Union	Mainx	HM035344*	
	*Dunaliella tertiolecta [this study]*	UTEX199	Brackish; salt lake, Soviet Union	Mainx	DQ157433	76097092
		UTEX199	Brackish; salt lake, Soviet Union	Mainx	DQ377086	87047577
*Dunaliella viridis *Massyuk		CONC002	Salar de Atacama, Chile	(1990)	AF313419	16596834
		CONC002	Salar de Atacama, Chile	(1990)	DQ377098	87047589
		SAG44.89			87047600	61200914
		SHU	China?		AY878700	58339343
		DCCBC4	Great Salt Lake, Utah, USA		AY686685	56578597
		DCCBC3	Great Salt Lake, Utah, USA		AY828227	61200913
Section *Peirceinae *((Halophilic species. Optimum salinity > 6% NaCl. Cells always green. Do not accumulate carotenes. Cells bilaterally symmetrical)						
*Dunaliella percei *Nicolai & Baas-Becking	*Dunaliella tertiolecta *[10,11]	CCAP19/2	Brackish; California, USA	Nicolai (1931)	HM035343*	
	*Dunaliella tertiolecta *[10,11]	UTEX2192	Brackish; California, USA	Nicolai (1931)	AF313443	16596846
Unknown *Dunaliella *Species						
*Dunaliella *sp.	*Dunaliella tertiolecta *[27]	CCAP19/23	Marine;	Pennick	HM035341*	
	*Dunaliella salina *[27]	CCAP19/12	Brackish; North Sinai, Israel	Ginzburg (1976)	HM035342*	
		CCMP367	Salt flat		DQ377087	87047578
		CCMP220	Salt flat		DQ377095	87047586
	*Dunaliella tertiolecta [this study]*	CCMP1923	Salt flat		DQ377094	87047585
	*Dunaliella tertiolecta [this study]*	CCMP1641	Salt flat		DQ377093	87047584
	*Dunaliella tertiolecta [this study]*	SAG19.6			AY582086	51035303
		FL1	Salt flat		DQ377099	87047590
	*Dunaliella viridis [this study]*	BSF1	USA: Utah, Bonneville Salt Flats	William Henley	DQ377081	87047572
	*Dunaliella viridis [this study]*	BSF2	USA: Utah, Bonneville Salt Flats	William Henley	DQ377082	87047573
	*Dunaliella viridis [this study]*	BSF3	USA: Utah, Bonneville Salt Flats	William Henley	DQ377083	87047574
	*Dunaliella salina [this study]*	006		A.W. Coleman, U. Brown	AF033278	2645739
	*Dunaliella tertiolecta [this study]*	hd10			DQ116747	71482602
	*Dunaliella viridis [this study]*	ABRIINW M1/1			EU927374	197290927
		ABRIINW M1/2	Iran?		EU927373	197290646
	*Dunaliella salina [this study]*	ABRIINW U1/1	Iran?		FJ164063	205371718
	*Dunaliella viridis [this study]*	ABRIINW U2/1	Iran?		FJ164064	205371719
		SPMO112-3	Salt flat, USA: Oklahoma, Salt Plains National Wildlife Refuge		DQ377103	87047594
		SPMO201-3	Salt flat, USA: Oklahoma, Salt Plains National Wildlife Refuge		DQ377110	87047601
		SPMO128-2	Salt flat, USA: Oklahoma, Salt Plains National Wildlife Refuge		DQ377105	87047596
		SPMO109-1	Salt flat, USA: Oklahoma, Salt Plains National Wildlife Refuge		DQ377105	87047596
		SPMO112-4	Salt flat, USA: Oklahoma, Salt Plains National Wildlife Refuge		DQ377104	87047595
		SPMO207-3	Salt flat, USA: Oklahoma, Salt Plains National Wildlife Refuge		DQ377115	87047606
		SPMO200-3	Salt flat, USA: Oklahoma, Salt Plains National Wildlife Refuge		DQ377107	87047598
		SPMO201-4	Salt flat, USA: Oklahoma, Salt Plains National Wildlife Refuge		DQ377111	87047602
		SPMO201-5	Salt flat, USA: Oklahoma, Salt Plains National Wildlife Refuge		DQ377112	87047603
		SPMO201-6	Salt flat, USA: Oklahoma, Salt Plains National Wildlife Refuge		DQ377113	87047604
		SPMO112-1	Salt flat, USA: Oklahoma, Salt Plains National Wildlife Refuge		DQ377101	87047592
		SPMO112-2	Salt flat, USA: Oklahoma, Salt Plains National Wildlife Refuge		DQ377102	87047593
		SPMO300-4	Salt flat, USA: Oklahoma, Salt Plains National Wildlife Refuge		DQ377118	87047609
		SPMO210-3	Salt flat, USA: Oklahoma, Salt Plains National Wildlife Refuge		DQ377116	87047607
	*Dunaliella viridis [this study]*	SPMO200-8	Salt flat, USA: Oklahoma, Salt Plains National Wildlife Refuge		DQ377108	87047599
	*Dunaliella viridis [this study]*	SPMO601-1	Salt flat, USA: Oklahoma, Salt Plains National Wildlife Refuge		DQ377121	87047612
	*Dunaliella viridis [this study]*	SPMO200-2	Salt flat, USA: Oklahoma, Salt Plains National Wildlife Refuge		DQ377106	87047597
	*Dunaliella viridis [this study]*	SPMO201-2	Salt flat, USA: Oklahoma, Salt Plains National Wildlife Refuge		DQ377109	87047600
	*Dunaliella viridis [this study]*	SPMO202-4	Salt flat, USA: Oklahoma, Salt Plains National Wildlife Refuge		DQ377114	87047605
	*Dunaliella viridis [this study]*	SPMO300-5	Salt flat, USA: Oklahoma, Salt Plains National Wildlife Refuge		DQ377119	87047610
	*Dunaliella viridis [this study]*	SPMO600-1	Salt flat, USA: Oklahoma, Salt Plains National Wildlife Refuge		DQ377120	87047611
	*Dunaliella viridis [this study]*	SPMO BP3	Salt flat, USA: Oklahoma, Salt Plains National Wildlife Refuge		DQ377122	87047613
	*Dunaliella viridis [this study]*	SPMO 980625-IE	Salt flat, USA: Oklahoma, Salt Plains National Wildlife Refuge		DQ377123	87047614
Other groups						
*Chlamydomonas reinhardtii*		SAG11-31			AJ749628	111073405
Chlamydomonas allensworthii		isolate 7			AF326855	13274452
*Oedogonium nodulosum *					DQ078301	71482662
*Bulbochaete rectangularis *var. *hiloensis *		UTEX954	Catawmont, Massachusetts, USA	Cook 1962	AY962677	62183531
*Astrephomene gubernaculifera*		UTEX2479			AGU66932	2982755
*Pandorina morum*		EP3			AF378359	14165185
*Eudorina elegans*		UTEX 1192			AF098173	4007503
*Yamagishiella unicocca*		UTEX2431			AF375785	14091689
*Phacotus lenticularis*		970805-20			AY009933	18025134
*Lobochlamys culleus*		UTEX1057	Maxville, Florida, USA	Smith	CCU66946	2627275

Asterisks indicate the strains sequenced in this study. Underlined strains correspond to identical isolates stored in different culture collections.

Acronyms: Culture Collection of Algae and Protozoa, UK (CCAP); Sammlug von Algenkulturen, Germany (SAG); University of Texas Culture Collection of Algae, USA (UTEX); American Type Culture Collection, USA (ATCC); Dunaliella Culture Collection at Brooklyn College, USA (DCCBC); Provasoli-Guillard National Centre for the culture of Marine Phytoplankton, USA (CCMP); Universidad de Concepción, Chile (CONC); Banco Canario de Algas, Spain (BCA) ; Instituto Tecnológico de Canarias, Spain (ITC).

Finally, the ITS2 data was not informative regarding the geographic origin of the *D. salina *strains.

## Discusion

The ITS2 Database allows automatic large scale simultaneous analyses of both ITS2 sequences and their secondary structures. Potential pitfalls are in structures obtained by different algorithms; however the main difficulty of performing a phylogenetic analysis of the genus *Dunaliella *is the misinformation available at Culture Collections and GenBank regarding the identification of strains and sequences. The tracking of the true identification of each strain was only possible after consulting several publications where the authors concluded that they were misidentified and suggested their re-identification [9,10,27]. In this study, we have also suggested the re-identification of some strains based on our ITS2 data. To overcome all these unnecessary problems (and given that it is unlikely that all the *Dunaliella *strains could be openly available), we suggest to establish a "type strain" for each *Dunaliella *taxon (including subspecies, forms or varieties). These basic data should be easily obtained from any official culture collection, thereby greatly facilitating comparison with new field isolates and avoiding misleading information and/or false conclusions.

Our ITS2 phylogenetic analysis of *Dunaliella *reveals several major groups, and positions the freshwater *D. lateralis *clearly outside *Dunaliella*, confirming that it no longer should be considered a member of this genus [10]. Nevertheless, the other freshwater species analyzed in this study (*D. acidophila*, CCAP19/35), maintained its position within the subgenus *Dunaliella*, and was not phylogenetically related to *D. lateralis*, as recently proposed [28]. Furthermore, the observation that different species belonging to several Sections (*Tertiolecta, Viridis, Dunaliella *and *Peircei*) share the exact same ITS2 sequence, make us believe that they correspond to a single species. These data agree with other authors [10,11,27,29], who suggested that the number of *Dunaliella *species may be much lower than it has been claimed till now. The possibility that the ITS2 gene is not able to discriminate between these species is highly unlikely; therefore, our observations support the suggestion that the morphological and physiological criteria available to discriminate *Dunaliella *species are either not very reliable [11], or are difficult to interpret..

In an attempt to clarify the species concept within *Dunaliella*, we searched for compensatory base changes (CBCs). Several case studies have revealed that the detection of a CBC in the ITS2 secondary structure between two organisms is correlated with sexual incompatibility [6,30,31], and these changes have been proposed as markers for distinguishing species [6,7,30,31]. In summary, these investigations conclude that while a CBC in a pair of sequences is positively correlated with species distinctness at a confidence level of 93%, the lack of a CBC in the ITS2 secondary structure does not necessarily indicate that two organisms belong to the same species [6]. The overall analysis of the CBC was not able to elucidate completely the species boundaries within the different groups of *Dunaliella*, since in some cases it was observed that there was a lack of CBCs between known distinct species.

In the special case of *D. salina*, high variation levels have been reported for decades [19-24]. However, only Massjuk [18] translated geographical, physiological, and morphological variables into the recognition of two subspecies (*D. salina *sp. *salina *and *D. salina *sp. *sibirica *Massjuk and Radch.) and three forms (*D. salina *sp. *salina *f. *salina, D. salina *sp. *salina *f. *oblonga *Lerche, and *D. salina *sp. *salina *f. *magna *Lerche). Later on, ITS2 phylogenetic analyses suggested the existence of two distinct phylogenetic species within the taxonomic entity currently known as *D. salina *[10,11,32], indicating the possibility of cryptic speciation [10]. Our ITS2 phylogenetic analysis does confirm the existence of three different groups within *D. salina*; however, the CBC results did not resolve if these groups may correspond to distinct species, although several strains of each *D. salina *group shared one CBC with *D. salina *strains in other groups. On the other hand, the high morphological and physiological variability found within the Spanish *D. salina *strains under the same lab conditions [24] was not correlated with the phylogenetic observations of this study. This finding indicates that the phylogenetic closeness found with ITS sequences does not reflect common physiological or morphological attributes. Moreover, our data unequivocally suggest that *D. salina *is not monophyletic, at odds with previous hypotheses [10,11,32].

The other objective of this work was to elucidate if the physiological uniqueness found in "Janubio" [such as its unique fatty acid profile and accumulation of high levels of carotenes under low light flux density conditions; Mendoza et al.: A new strategy for carotenogenesis under conditions of cellular stress in *Dunaliella *(a Potential New Species), submitted] could be confirmed by the ITS2 data and the CBC analysis. However, although we observed that this strain has a unique ITS2 sequence profile, and had more than one CBC with the other phylogenetically related *D. salina*, our data do not allow us to conclude that this is a new species, and further studies must be performed to find out if the differences observed are just reflecting a high intra-specific variability. Finally, in agreement with previous studies [8,11,25], our ITS2 data failed to furnish evidence for isolation by distance among *D. salina *strains.

## Conclusion

This work demonstrates that the taxonomy of *Dunaliella *should be revised. The great diversity observed within the ITS2 sequences of *D. salina *suggests that different biological groups may exist within this taxon; however, this was not confirmed with the CBC analysis. Likewise, although the Spanish *D. salina *strain ITC5105 "Janubio" was characterized by a unique ITS2 sequence, the hypothesis that it may be a new species could not be confirmed by the CBCs analysis, requiring further morpho-physiological and genetic investigation. Overall, the use of CBCs to define species boundaries within *Dunaliella *was not conclusive in some of the cases assessed.

## Methods

### Strains, DNA extraction and ITS amplification

We sequenced the ITS2 region of 13 *D. salina *strains from Spanish and French saltworks, one strain obtained from the Culture Collection of Algae and Protozoa UK (CCAP), and one *D. salina *strain that has been maintained in the Instituto Tecnológico de Canarias (ITC henceforth) for several years (purchased from CCAP as *Dunaliella salina *19/30). We also sequenced other *Dunaliella *species (*D. minuta *CCAP19/5, *D. tertiolecta *CCAP19/23 and CCAP19/6B, *D. bioculata *CCAP19/4) [Table 1]. The sequences of the other strains analyzed in this study were obtained from the ITS2 Database (http://its2.bioapps.biozentrum.uni-wuerzburg.de/?about). Detailed information about the strains used in this study can be found in Table 1.

DNA extraction was performed with a chelex-100 (Biorad, CA, USA) resin-based protocol [33]. For the DNA amplification of the ITS region, primers AB28 and TW81 in Goff et al. (1994) [34] were used. DNA amplification was carried out in a total volume of 25 μl with 1X iQ SYBR Green Supermix (Biorad, CA, USA) and 10 pM of each primer in a Smart Cycler thermocyler (Cepheid,CA, USA) as follows: 5 min at 94°C; 5 cycles of 1 min at 94°C, 2 min at 50°C and 1 min at 72°C; 30 cycles of 1 min at 94°C, 1 min at 62°C and 1 min at 72°C, with a final extension of 5 min at 72°C.

PCR products were first electrophoresed in a 1.5% agarose gel to assure that a single band of 500-600 bp was present, then purified using the Real Clean Spin kit (REAL, Durviz S.L.U., Valencia, Spain), and finally bi-directionally sequenced on an ABI PRISM 3730xl automatic sequencer (Applied Biosystems, CA, USA) at the DNA sequencing services of Macrogen (Korea).

### Phylogenetic analyses

Sequences and their individual secondary structures were obtained from the ITS2 Database [35-37]. Newly obtained ITS2 sequences were annotated according to Keller et al. [38], and their secondary structures predicted by homology modeling [39]. The phylogenetic analysis followed the procedure outlined in Schultz and Wolf [7] in accordance with Keller et al. [5]. The software used for the ITS2 sequence-structure analysis can be obtained from http://its2.bioapps.biozentrum.uni-wuerzburg.de/?about. A global, multiple sequence-structure alignment was generated in 4SALE v1.5 [40,41]. The sequences and their individual secondary structures were synchronously aligned making use of an ITS2 sequence-structure specific scoring matrix [40], and the start and end of the alignment was manually adjusted. Based on primary and secondary structure information, phylogenetic relationships were reconstructed by ProfDistS, through the use of an ITS2 specific, general time reversible substitution model [42,43]. Bootstrap support [44] was estimated on 100 pseudo-replicates. The resulting tree was visualized with TreeView [45].

To study the species boundaries within *Dunaliella *we followed the "distinguishing species" instructions [6] based on compensatory base changes (CBCs) in the ITS2 secondary structure, and we used the CBCAnalyzer option implemented in 4SALE.

## Competing interests

The authors declare that they have no competing interests.

## Authors' contributions

PA carried out the laboratory work, the phylogenetic analysis and wrote the manuscript. RJ-M and JC-C helped with the phylogenetic analysis and revised the manuscript. HM, AJ, LC and KF isolated the *Dunaliella *strains and revised the manuscript. HM conceived the study. All authors have read and approved the final manuscript.

## Supplementary Material

Additional file 1 **Compensatory Base Changes (CBC) analysis**. Compensatory Base Changes (CBCs) between different groups and species within the *Dunaliella *taxon (Excel file). Click here for file
